# RACK1 and NEK7 mediate GSDMD-dependent macrophage pyroptosis upon *Streptococcus suis* infection

**DOI:** 10.1186/s13567-024-01376-w

**Published:** 2024-09-27

**Authors:** Xin Shen, Jinrong Ran, Qingqing Yang, Bingjie Li, Yi Lu, Jiajia Zheng, Liuyi Xu, Kaixiang Jia, Zhiwei Li, Lianci Peng, Rendong Fang

**Affiliations:** Joint International Research Laboratory of Animal Health and Animal Food Safety, College of Veterinary Medicine, Southwest University, Chongqing, 400715 China

**Keywords:** *Streptococcus suis*, pyroptosis, NLRP3 inflammasome, GSDMD, RACK1, NEK7

## Abstract

*Streptococcus suis* serotype 2 (SS2) is an important zoonotic pathogen that induces an NLRP3-dependent cytokine storm. NLRP3 inflammasome activation triggers not only an inflammatory response but also pyroptosis. However, the exact mechanism underlying *S. suis*-induced macrophage pyroptosis is not clear. Our results showed that SS2 induced the expression of pyroptosis-associated factors, including lactate dehydrogenase (LDH) release, propidium iodide (PI) uptake and GSDMD-N expression, as well as NLRP3 inflammasome activation and IL-1β secretion. However, GSDMD deficiency and NLRP3 inhibition using MCC950 attenuated the SS2-induced expression of pyroptosis-associated factors, suggesting that SS2 induces NLRP3-GSDMD-dependent pyroptosis. Furthermore, RACK1 knockdown also reduced the expression of pyroptosis-associated factors. In addition, RACK1 knockdown downregulated the expression of NLRP3 and Pro-IL-1β as well as the phosphorylation of P65. Surprisingly, the interaction between RACK1 and P65 was detected by co-immunoprecipitation, indicating that RACK1 induces macrophage pyroptosis by mediating the phosphorylation of P65 to promote the transcription of NLRP3 and pro-IL-1β. Similarly, NEK7 knockdown decreased the expression of pyroptosis-associated factors and ASC oligomerization. Moreover, the results of co-immunoprecipitation revealed the interaction of NEK7-RACK1-NLRP3 during SS2 infection, demonstrating that NEK7 mediates SS2-induced pyroptosis via the regulation of NLRP3 inflammasome assembly and activation. These results demonstrate the important role of RACK1 and NEK7 in SS2-induced pyroptosis. Our study provides new insight into SS2-induced cell death.

## Introduction

*Streptococcus suis* (*S. suis*) is an important zoonotic pathogen that causes different diseases, including arthritis, sepsis, septic shock, and meningitis, in pigs and humans [1]. Currently, 29 serotypes of *S. suis* exist, of which serotype 2 (SS2) is the most virulent and widely distributed strain [2]. Notably, two large-scale outbreaks of SS2 with streptococcal toxic shock-like syndrome (STSLS) occurred on pig farms in China in 1998 and 2005 and led to 14 deaths in Jiangsu and 38 deaths in Sichuan, respectively, which threatens global public health [3, 4]. Virulence factors, such as capsular polysaccharide (CPS), suilysin (SLY), enolase and muramidase-released protein (MRP), have been characterized to reveal the pathogenic mechanism of SS2 infection [5]. In addition, the study of the interaction between the host and SS2 contributes to revealing the pathogenesis mechanism involved.

Inflammation is the hallmark of SS2 infection. SS2 has been reported to induce streptococcal toxic shock-like syndrome (STSLS) with a cytokine storm through the activation of the NLRP3 inflammasome, leading to the secretion of IL-1β and IL-18 [6]. Notably, the release of IL-1β is mediated by caspase-1-cleaved gasdermin D (GSDMD), which regulates a cell death mode termed pyroptosis. Pyroptosis is a form of programmed inflammatory cell death that can be triggered by pathogens and plays a critical role in the clearance of microbial infection and warning of endogenous risk signals [7].

Upon activation of the NLRP3 inflammasome, activated caspase-1 cleaves GSDMD to form GSDMD-N, which binds to phosphatidylinositol phosphates and phosphatidylserine in the inner leaflet of the cell membrane to induce the formation of membrane pores [8, 9], triggering pyroptosis during various bacterial infections, including infections with *E. coli*, *Staphylococcus pseudintermedius*, *Salmonella* and *Staphylococcus aureus* [10–13]. SS2 has been reported to induce cell apoptosis, which is mediated by caspase-3 and caspase-8 [14]. A recent study revealed that SS2-derived membrane vesicles induce GSDMD-mediated endothelial cell pyroptosis [15]. However, the exact mechanism underlying *S. suis*-induced macrophage pyroptosis is not fully understood.

RACK1 is a highly conserved member of the tryptophan‒aspartic acid (WD) repeat protein family [16] and is known as a multifunctional scaffold protein involved in many biological processes, including proliferation, cell migration, apoptosis and development [17, 18]. In addition, RACK1 plays an important role in microbial infection [19, 20]. Furthermore, RACK1 has been shown to interact with NLRP3 to mediate NLRP3 inflammasome activation during microbial infection [21]. Importantly, RACK1 is involved in the regulation of the NLRP3-caspase-1-GSDMD-IL-1β signalling pathway during *Mycobacterium tuberculosis* infection [22], indicating that RACK1 plays an important role in pyroptosis.

In the present study, we explored RACK1-mediated *S. suis*-induced pyroptosis in macrophages. The results showed that *S. suis* infection activated the NLRP3 inflammasome and markedly caused cell death via the NLRP3/GSDMD signalling pathway. Further study revealed that RACK1 bound to the NF-κB subunit P65 to regulate the phosphorylation of P65 and then reduced the expression of NLRP3 and Pro-IL-1β, leading to the inhibition of NLRP3 inflammasome activation and a reduction in GSDMD. Finally, knockdown of NEK7 markedly inhibited ASC oligomerization and GSDMD expression, suggesting that NEK7 serves as a critical protein that mediates *S. suis*-induced pyroptosis by promoting NLRP3 inflammasome assembly and activation. Our findings provide a better understanding of the mechanism of *S. suis*-induced pyroptosis, which provides a potential therapeutic target for treating *S. suis* infection.

## Materials and methods

### Bacterial strains

The *S. suis* P1/7 (serotype 2) used in the present study was kindly provided by Prof. Xiangru Wang (College of Veterinary Medicine, Huazhong Agricultural University). P1/7 was grown at 37 ℃ in Todd Hewitt broth (THB; BBL Microbiology Systems, Cockeysville, MA, USA). Bacterial concentrations were quantified by a colony counting assay, and bacteria were diluted with cell culture medium to the indicated concentrations for experimental use.

### Mice

Wild-type (WT) C57BL/6 mice were purchased from the Chongqing Academy of Chinese Material Medical (Chongqing, China). Gsdmd^−/−^ mice were kindly provided by Dr. Feng Shao from the National Institute of Biological Sciences (Beijing, China). These knockout mice were on a C57BL/6 background. All the mice were maintained in specific pathogen-free (SPF) conditions for use at 8–10 weeks of age. All the animal experiments were approved by the Institutional Animal Care and Use Committee (IACUC) of Southwest University, Chongqing, China (IACUC-20210215-05).

### Preparation of cells and *S. suis* infection

Mouse primary peritoneal macrophages (PECs) were collected as previously reported [23]. Briefly, the mice were intraperitoneally injected with 2 mL of 4% thioacetate (Aiken, Tokyo, Japan). After 3‒4 days, PECs were harvested from peritoneal exudates and resuspended in RPMI 1640 with 10% (v/v) heat-inactivated FBS (Gibco). The cells were seeded at 1 × 10^6^ cells/well in 12-well plates, 2 × 10^5^ cells/well in 48-well plates or 1 × 10^5^ cells/well in 96-well plates. These cells were incubated in a humidified 37 ℃ incubator with 5% CO_2_. After 2 h of incubation, the nonadherent cells were removed, and the adherent cells were used for the assays described below.

WT and Gsdmd^−/−^ PECs were infected with P1/7 at a multiplicity of infection (MOI) of 5. After 6 h of infection, gentamycin (250 μg/mL, Solarbio, China) was added for an additional 18 h. Then, the supernatants and cell lysates were collected and used in the assays described below. In some experiments, the cells were pretreated with various inhibitors, including MCC950 (10 μM), Z-VAD-FMK (5 μM) and BAY (10 μM), for 1 h.

### Enzyme-linked immunosorbent assay (ELISA)

The cells were infected with P1/7 as described above. After infection, the supernatants and cell lysates were collected to determine the concentrations of cytokines, including IL-1β and IL-6 (Invitrogen, CA, USA), by ELISA according to the manufacturer’s instructions.

### Lactate dehydrogenase (LDH) cytotoxicity assay

The cells were seeded in a 96-well plate as described above. After infection, the supernatants were collected for cytotoxicity analysis via the Lactic Dehydrogenase Release Assay Kit (C0017, Beyotime) according to the manufacturer’s instructions. Finally, the absorbance was measured at 490 nm with a microplate reader (Bio-Rad, Japan) and was corrected for absorbance at 630 nm.

### SiRNA transfection

The cells were prepared as described above and transfected with siRNA by Lipofectamine 3000 (Thermo Fisher Scientific, USA) for 48 h. The siRNA sequences used in the present study were as follows: RACK1 siRNA (Sangon Biotech, 5′-CCACAAUGGAUGGGUAACACATT-3′), NEK7 siRNA (Sangon Biotech, 5′-GAUAGACUGUGUUUAUAGATT-3′) and control siRNA (Sangon Biotech, 5′-UUCUCCGAACGUGUCACGUTT-3′). After transfection, the cells were infected with P1/7 as described above. Finally, the cell supernatants and lysates were collected for ELISA, western blot and RT‒PCR analysis.

### Propidium iodide (PI) staining

PECs were prepared in 48-well plates and infected with P1/7 as described above. After infection, the cells were stained with Hoechst/PI (KeyGEN BioTECH, Jiangsu, China). After staining, the cells were observed by fluorescence microscopy (Olympus, Tokyo, Japan).

### Western blot analysis

PECs were prepared in 12-well plates and infected with P1/7 as described above. The supernatants were subsequently collected and concentrated with 20% (w/v) trichloroacetate (TCA), after which the cells were lysed with 1 × SDS loading buffer (Beyotime, Beijing, China). Next, the supernatants and cell lysates were subjected to 10–15% SDS‒PAGE and then transferred to polyvinylidene difluoride (PVDF) membranes by electroblotting. The membranes were blocked with 5% nonfat dry milk and then immunoblotted with the indicated antibodies (Abs), including anti-GSDMD Ab (Abcam, Cambridge, UK), anti-NLRP3 Ab (Cell Signaling Technology, USA), anti-caspase-1 Ab (AdipoGen, San Diego, USA), anti-IL-1β Ab (Bioss, Beijing, China), anti-ASC Ab (Santa Cruz Biotechnology, Inc), anti-NEK7 Ab (Abcam, Cambridge, UK), anti-RACK1 Ab (Cell Signaling Technology, USA), anti-NF-κB P65 Ab (Beyotime, Beijing, China), anti-phospho-NF-κB P65 Ab (Proteintech, China), anti-ERK Ab (Bioss Beijing, China), anti-phospho-ERK Ab (Cell Signaling Technology, USA), anti-JNK Ab (Cell Signaling Technology, USA), anti-phospho-JNK Ab (Bioss, Beijing, China) and anti-β-actin Ab (Beyotime, Beijing, China). Finally, distinct protein bands were detected with an enhanced chemiluminescence (ECL) detection reagent (Biosharp, China).

### ASC oligomerization

PECs were prepared in 12-well plates and infected with P1/7 as described above. After the indicated time of infection, the cells were lysed with cold PBS containing 0.5% Triton X-100, and the cell lysates were centrifuged at 13 000 rpm for 15 min at 4 ℃ to obtain cell pellets. Then, the pellets were washed twice with cold PBS and suspended in 200 μL of PBS. The resuspended pellets were subsequently crosslinked with 2 mM fresh disuccinimidyl suberate (DSS) at 37 ℃ for 30 min, after which the pellets were centrifuged at 13 000 rpm for 15 min at 4 ℃. Finally, the cross-linked pellets were dissolved in 30 μL of 1 × SDS‒PAGE sample loading buffer, and the samples were boiled for 5 min before western blot analysis.

### Quantitative RT‑PCR

PECs were infected with P1/7 as described above. After infection, total RNA was extracted from the cells using TRIzol Reagent (Life Technologies Carlsbad, CA, USA) according to the manufacturer’s protocols. Complementary DNA was generated using the PrimeScript^®^ RT Reagent Kit (TaKaRa, Dalian, China) according to the manufacturer’s instructions. Subsequently, quantitative real-time PCR (RT‒PCR) was performed using the CFX96 RT‒PCR detection system (Bio-Rad, United States). The primers used in this study are as follows: β-actin forward, 5′-GTGACGTTGACATCCGTAAAGA-3′ and reverse, 5′-GCCGGACTCATCGTACTCC-3′; RACK1 forward, 5′-ACTCCCACTTCGTTAGTGATGT-3′ and reverse, 5′-CCCGTTGTGAGATCCCAGAG-3′; and NEK7 forward, 5′-AAAGAGGCTAATCCCTGAGAGAA-3′ and reverse, 5′-CTACCCCAGTGGCTGTAATGA-3′. The reaction procedure was as follows: 95 ℃, 2 min; 95 ℃, 5 s, 40 cycles; 60 °C, 30 s; 95 ℃, 5 s; 60 ℃, 5 s; 95 ℃, 2 min. Relative gene expression levels were normalized against the expression levels of β-actin.

### Coimmunoprecipitation analysis

PECs were prepared and infected with P1/7 as described above. After infection, the cells were lysed at 4 ℃ in ice-cold cell lysis buffer, followed by centrifugation at 12 000 rpm for 15 min at 4 ℃. The supernatants were subsequently collected and incubated with 1–2 μg of the indicated antibody overnight at 4 ℃. Next, protein A + G agarose beads (Beyotime, Beijing, China) were added to the lysates and incubated for 2 h at 4 ℃ to pull down target proteins that bound to the antibodies. The beads were subsequently washed 4 times with lysis buffer, boiled in 1 × SDS loading buffer at 100 ℃ for 5 min and subjected to immunoblotting analysis.

### Statistical analysis

All the data are presented as the mean ± SEM of three independent experiments for each group (*n* = 3). One-way ANOVA was used to analyse the statistical significance among different groups. All the graphs were generated using GraphPad Prism software (San Diego, CA, USA). Statistical significance is shown as *p < 0.05, ** p < 0.01, ***p < 0.001, ns = not significant.

## Results

### P1/7 infection induces macrophage pyroptosis

**Figure 1:**
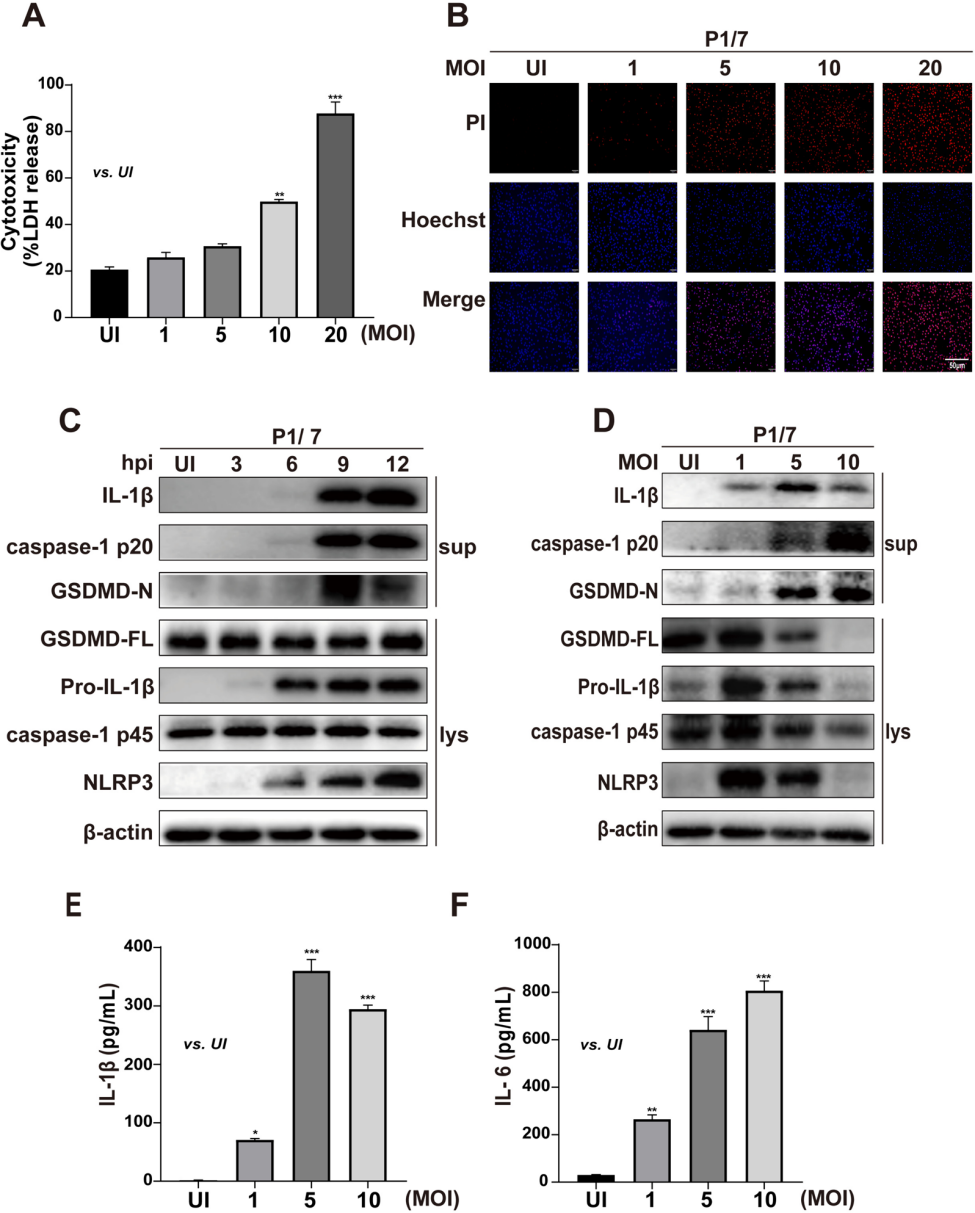
**P1/7 infection induces macrophage pyroptosis**. Primary peritoneal macrophages (PECs) were infected with P1/7 at MOIs of 1, 5, 10 and 20 for the indicated times. Lactate dehydrogenase (LDH) release (**A**) and propidium iodide (PI) uptake (**B**) were used to determine cell death. Western blotting was used to detect the protein levels of NLRP3, caspase-1, GSDMD and IL-1β (**C, D**). ELISA was used to determine the secretion levels of IL-1β and IL-6 (**E, F**). The data are presented as the mean ± SEM of three independent experiments with triplicate samples per experiment. *p < 0.05, **p < 0.01, ***p < 0.001.

Pyroptosis is an inflammatory form of programmed cell death that can be triggered by invading pathogens [24]. A recent study reported that *S. suis* induces cell death, but it is not clear which types of cell death are involved in *S. suis* infection. We investigated whether *S. suis* strain P1/7 induced pyroptosis in primary mouse peritoneal macrophages via LDH release, PI staining and NLRP3 inflammasome activation. P1/7 significantly induced LDH release in macrophages in an MOI-dependent manner (Figure 1A). Similarly, P1/7 significantly triggered PI influx in macrophages as the red fluorescent signal increased (Figure 1B), indicating that P1/7 induced cell death. Furthermore, P1/7 induced NLRP3 inflammasome activation with increased protein expression of NLRP3 and caspase-1 at 9 h post-infection (hpi) (Figures 1C, D), leading to the production of inflammatory cytokines, including IL-1β and IL-6 (Figures 1E, F). Importantly, P1/7 induced the secretion of mature GSDMD (GSDMD-N) in an MOI-dependent manner (Figure 1D). These results demonstrate that P1/7 infection activates the NLRP3 inflammasome and caspase-1, leading to the formation of mature GSDMD, which results in inflammatory pyroptosis in macrophages.

### P1/7 triggers macrophage pyroptosis and IL-1β secretion via NLRP3 and GSDMD

**Figure 2:**
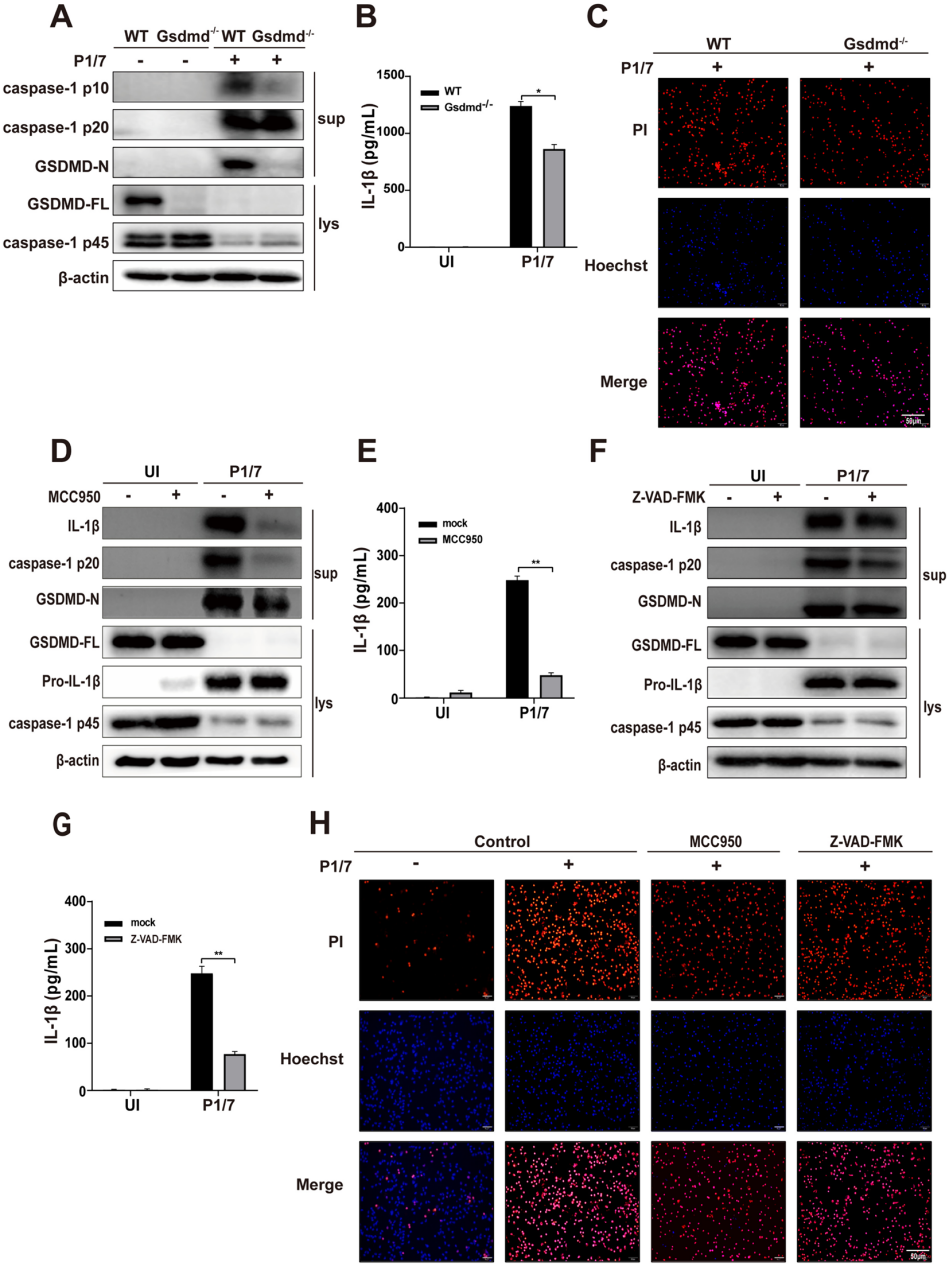
**P1/7 triggers macrophage pyroptosis and IL-1β secretion via NLRP3 and GSDMD**. PECs from WT (C57BL/6) or Gsdmd^−/−^ (C57BL/6) mice were uninfected or infected with P1/7 (MOI = 5) for 24 h. Western blotting was used to detect the expression of GSDMD and caspase-1 (**A**). ELISA was used to determine IL-1β secretion (**B**). PI uptake by PECs from WT and Gsdmd^−/−^ mice was detected (**C**). In addition, PECs were pretreated with MCC950 (10 μM) for 1 h prior to infection. After infection, western blotting was used to detect the protein levels of caspase-1, GSDMD and IL-1β (**D**), and ELISA was used to determine the secretion of IL-1β (**E**). Furthermore, PECs were pretreated with Z-VAD-FMK (5 μM) for 1 h prior to infection. After infection, the protein levels of caspase-1, GSDMD and IL-1β (**F**) and the secretion of IL − 1β were determined (**G**). Moreover, images of PI uptake are shown (**H**). The data are presented as the mean ± SEM of three independent experiments with triplicate samples per experiment. *p < 0.05, **p < 0.01.

GSDMD plays an important role in inducing pyroptosis, and the N-terminal fragment of GSDMD forms a polymeric pore in the cell membrane, causing pyroptosis [25]. Therefore, Gsdmd^−/−^ mouse peritoneal macrophages were used to investigate the role of GSDMD in *S. suis*-induced pyroptosis. Our results revealed that GSDMD deficiency markedly reduced the activation of caspase-1 (caspase-1 p10) and IL-1β secretion in P1/7-infected cells (Figures 2A and B). Furthermore, the results of PI staining revealed that GSDMD deficiency markedly inhibited P1/7-induced cell death in P1/7-infected cells (Figure 2C). These results indicate that GSDMD plays an essential role in P1/7-induced inflammatory pyroptosis.

NLRP3 inflammasome activation induces activated caspase-1 cleavage of GSDMD to promote IL-1β secretion. To further investigate whether NLRP3 inflammasome activation is involved in P1/7-induced pyroptosis, cells were pretreated with the NLRP3 inflammasome inhibitor MCC950 prior to infection. As shown in Figures 2D and E, MCC950 reduced the expression of GSDMD-N, caspase-1 (p20) and IL-1β (Figure 2E), indicating that P1/7-induced inflammatory pyroptosis is dependent on NLRP3. Similarly, the caspase inhibitor Z-VAD-FMK inhibited the cleavage of GSDMD and IL-1β production during P1/7 infection (Figures 2F and G). Furthermore, the results of PI staining revealed that MCC950 and Z-VAD-FMK inhibited P1/7-induced macrophage death (Figure 2H). Together, these results demonstrate that P1/7 triggers macrophage pyroptosis via the NLRP3/GSDMD/caspase-1/11 pathway.

### RACK1 mediates P1/7-induced macrophage pyroptosis

**Figure 3:**
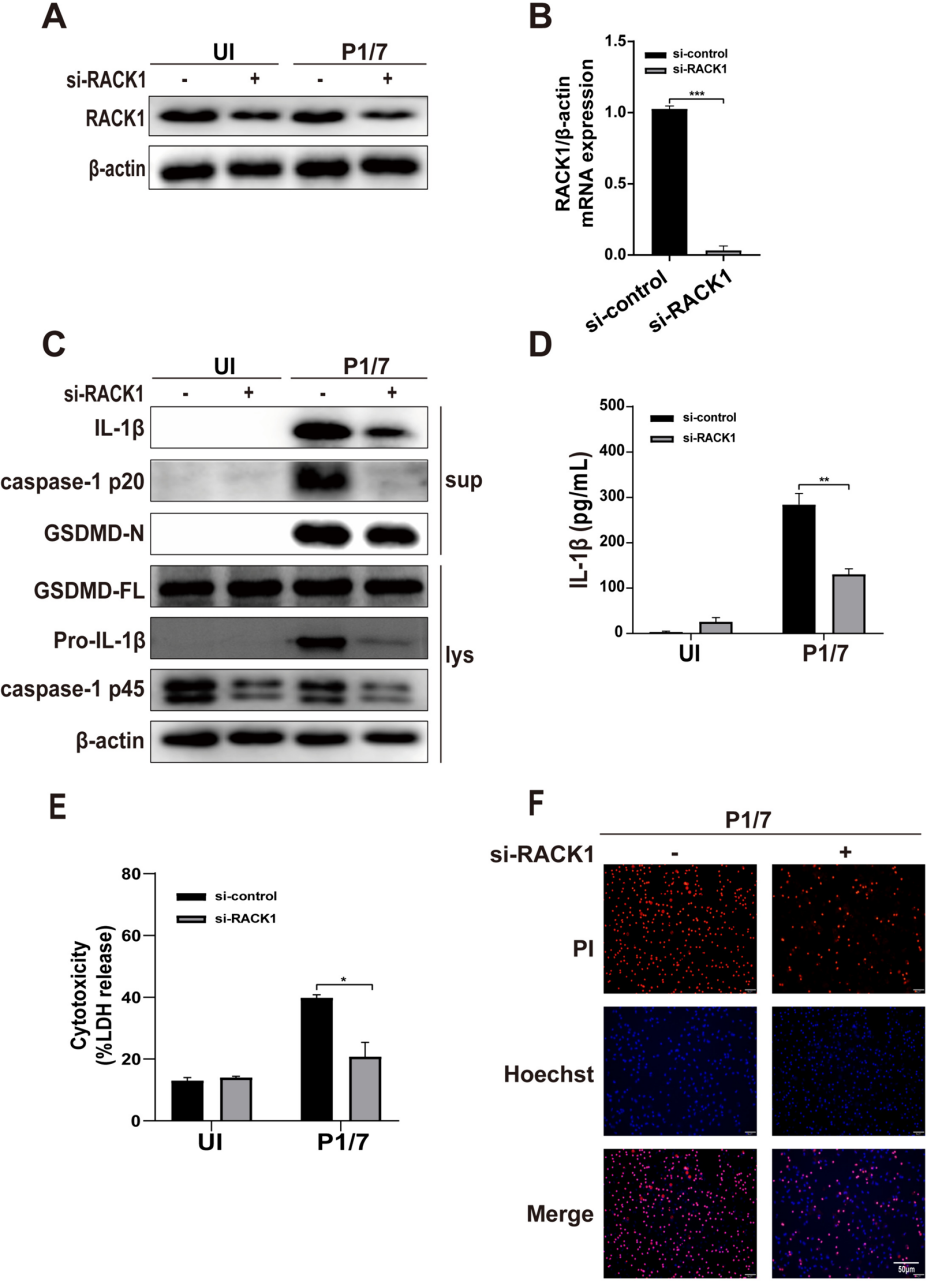
**RACK1 mediates P1/7-induced macrophage pyroptosis**. PECs were transfected with siRNA for 48 h to knock down RACK1. After transfection, western blotting and RT‒PCR were used to detect RACK1 protein expression (**A**) and RACK mRNA expression (**B**). PECs were transfected with RACK1 siRNA and then infected with P1/7 for 24 h. The protein levels of caspase-1, GSDMD and IL-1β in RACK1-knockdown macrophages were measured by western blotting (**C**). The level of secreted IL-1β was measured by ELISA (**D**). Macrophage death was measured by LDH release (**E**) and PI uptake (**F**) in RACK1-knockdown macrophages. The data are presented as the mean ± SEM of three independent experiments with triplicate samples per experiment. *p < 0.05, **p < 0.01, ***p < 0.001.

It has been reported that RACK1 can interact with NLRP3 and promote its activation [26]. To investigate the role of RACK1 in P1/7-induced pyroptosis, siRNA was used to knockdown RACK1 in macrophages. The results of western blot and RT‒PCR revealed that si-RACK1 significantly reduced the protein and mRNA expression of RACK1 (Figures 3A and B), demonstrating successful knockdown of RACK1 in macrophages. Knockdown of RACK1 reduced the cleavage of caspase-1 and GSDMD as well as IL-1β production in P1/7-infected macrophages (Figures 3C and D). Consistently, RACK1 knockdown significantly reduced LDH release (Figure 3E) and PI influx (Figure 3F), suggesting that RACK1 is a critical mediator of P1/7-induced pyroptosis.

### RACK1 mediates P1/7-induced macrophage pyroptosis via the phosphorylation of P65

**Figure 4:**
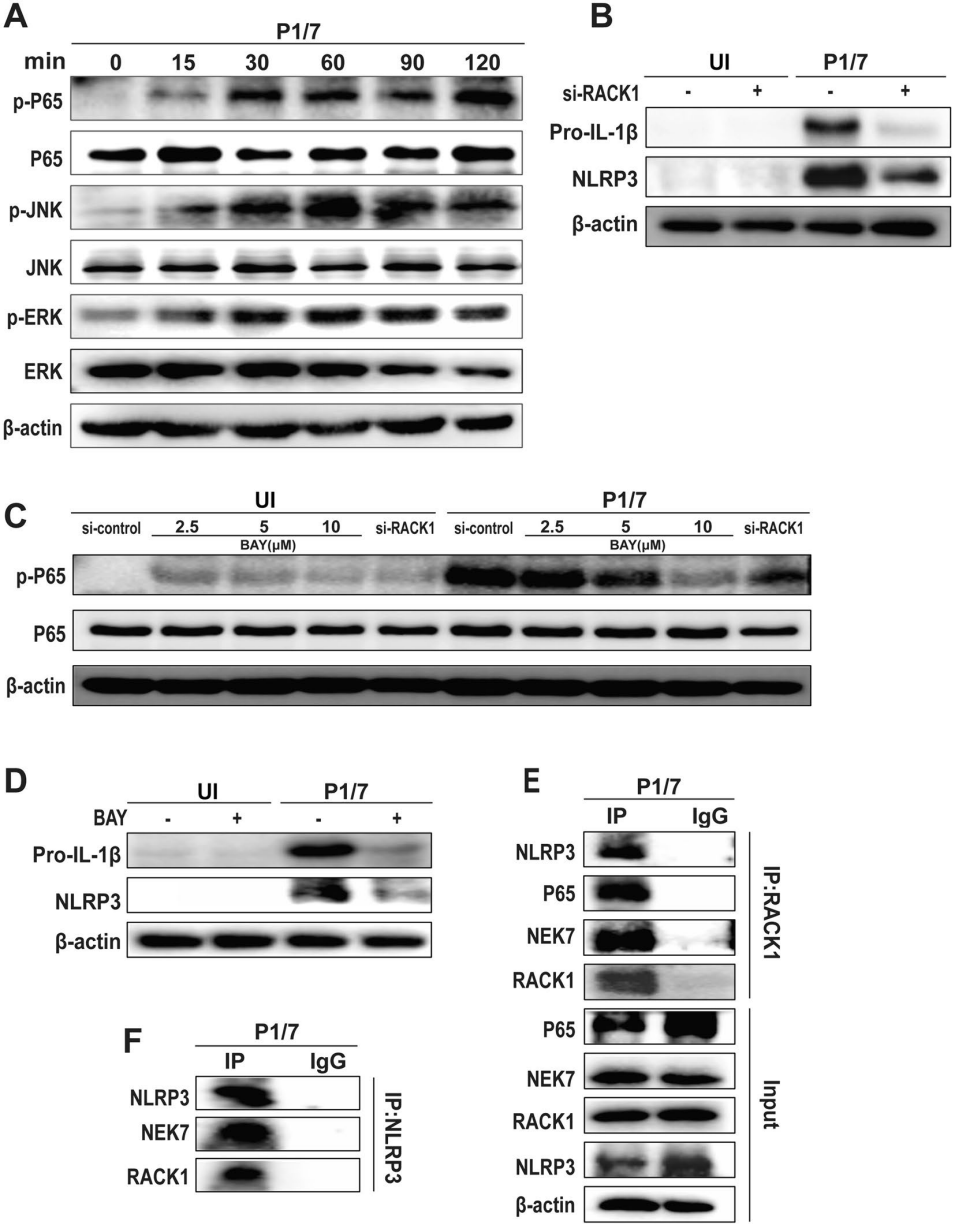
**RACK1 mediates P1/7-induced macrophage pyroptosis via the phosphorylation of P65**. PECs were infected with P1/7 (MOI = 5) for the indicated times. After infection, the protein expression of P65, p-P65, ERK, p-ERK, JNK and p-JNK was measured by western blotting (**A**). PECs were transfected with si-RACK1 and then infected with P1/7 for 9 h. After infection, the protein expression of NLRP3 and Pro-IL-1β was measured by western blotting (**B**). The protein expression of P65 and p-P65 in P1/7-infected cells was measured after treatment with BAY and the knockdown of RACK1 at 2 h (**C**). PECs were pretreated with BAY (10 μM) for 1 h and then infected with P1/7 for 9 h. The protein expression of NLRP3 and Pro-IL-1β was measured (**D**). Immunoprecipitation with anti-RACK1 (**E**) and anti-NLRP3 (**F**) antibodies was performed. The data are presented as the mean ± SEM of three independent experiments with triplicate samples per experiment.

Inflammatory pyroptosis can be induced by the activation of the NLRP3 inflammasome, which is controlled by multiple upstream signalling pathways [27, 28]. To investigate the mechanism by which RACK1 mediates P1/7-induced pyroptosis, the upstream signalling pathways were detected. As shown in Figure 4A, P1/7 significantly increased the levels of phosphorylated extracellular signal-regulated kinase (ERK), nuclear factor κB (NF-κB) P65, and c-Jun N-terminal kinase (JNK) with infection time. However, RACK1 knockdown significantly abrogated P1/7-induced protein expression of Pro-IL-1β and NLRP3 (Figure 4B), suggesting that RACK1 might regulate the transcription of Pro-IL-1β and NLRP3. Furthermore, RACK1 knockdown significantly decreased the P1/7-induced phosphorylation of P65, which is similar to the effect of treatment with the NF-κB inhibitor BAY (Figure 4C), suggesting that RACK1 mediates the phosphorylation of P65 during P1/7 infection. In addition, BAY treatment significantly inhibited the expression of NLRP3 and Pro-IL-1β (Figure 4D), suggesting that RACK1 mediates the activation of the NLRP3 inflammasome via NF-κB activation.

To further investigate the role of RACK1 in P1/7-infected macrophages, we immunoprecipitated cell lysates from macrophages with an anti-RACK1 antibody, and the results revealed that RACK1 interacted with P65 in P1/7-infected macrophages (Figure 4E). Unsurprisingly, the immunoprecipitation results also revealed that RACK1 interacted with NLRP3 and NEK7 in P1/7-infected macrophages (Figure 4E). Moreover, the results of P1/7-infected cells immunoprecipitated with an anti-NLRP3 antibody also revealed that RACK1 interacted with NLRP3 and NEK7 (Figure 4F). These results suggest that RACK1 plays an important role in P1/7-induced activation of NF-κB and the NLRP3 inflammasome.

### NEK7 is involved in P1/7-induced macrophage pyroptosis

**Figure 5:**
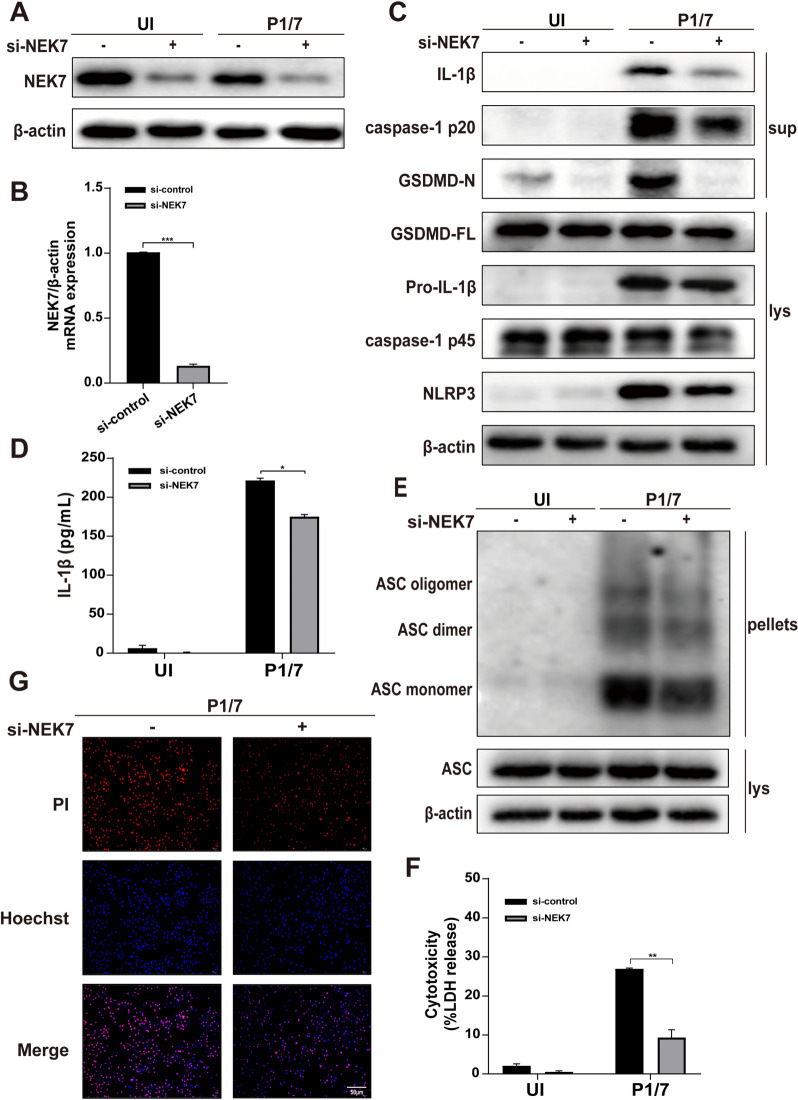
**NEK7 is involved in P1/7-induced macrophage pyroptosis**. PECs were transfected with si-NEK7 for 48 h. After transfection, western blotting and RT‒PCR were used to detect NEK7 protein expression (**A**) and NEK7 mRNA expression (**B**). The protein levels of NLRP3, caspase-1, GSDMD and IL-1β in NEK7-knockdown macrophages were detected during P1/7 infection (**C**). The level of secreted IL-1β is shown in NEK7-knockdown cells during P1/7 infection (**D**). ASC oligomerization was detected in si-NC-transfected and si-NEK7-transfected cells during P1/7 infection (**E**). Cell death was measured by LDH release (**F**) and PI uptake (**G**). The data are presented as the mean ± SEM of three independent experiments with triplicate samples per experiment. *p < 0.05, **p < 0.01, ***p < 0.001.

NEK7 has been reported to play an essential role in the activation of the NLRP3 inflammasome [29]. Importantly, the interaction of NEK7 and NLRP3 modulates pyroptosis [30]. Our results showed that P1/7 induced the interaction of NLRP3, NEK7 and RACK1 (Figure 4F). Therefore, we further investigated the effect of NEK7 on P1/7-induced pyroptosis using siRNA-mediated knockdown of NEK7. As shown in Figure 5A and B, siRNA significantly decreased the protein and mRNA expression of NEK7, demonstrating that NEK7 was successfully disrupted in macrophages. Notably, the knockdown of endogenous NEK7 resulted in decreased protein expression of NLRP3, IL-1β, caspase-1 (p20), and GSDMD-N in P1/7-infected macrophages (Figure 5C). In addition, the knockdown of NEK7 inhibited the secretion of IL-1β (Figure 5D). Notably, the knockdown of NEK7 suppressed the oligomerization of ASC (Figure 5E), which is involved in the activation and assembly of the NLRP3 inflammasome. Furthermore, NEK7 knockdown significantly reduced LDH release and PI influx in P1/7-infected macrophages (Figures 5F, G). Taken together, these findings indicate that NEK7 promotes P1/7-induced pyroptosis by mediating the assembly and activation of the NLRP3 inflammasome.

## Discussion

*S. suis* is an important zoonotic pathogen that interacts with the host to induce the evolution of diverse mechanisms to escape attack by immune cells, such as the ability to cleave the human antimicrobial peptide LL-37 to avoid being killed by neutrophils and to avoid being phagocytosed by immune cells [31, 32]. Among immune cells, macrophages play a critical role in killing invading pathogens. However, *S. suis* has developed diverse mechanisms to avoid phagocytosis, such as the existence of CPS [33]. Furthermore, the induction of cell death, including cell apoptosis and pyroptosis, is another mechanism by which *S. suis* attacks the immune system of the host [14]. It has been reported that some virulence factors of *S. suis,* including PrsA and its membrane vesicles, induce pyroptosis [34]. However, the exact mechanism of *S. suis*-induced pyroptosis is still not clear.

Pyroptosis, as a form of programmed cell death, could provide danger signals for the host to generate a protective inflammatory response against microbial infection, but excessive inflammatory pyroptosis causes pathological injury in the host [35]. Thus, pyroptosis might be a double-edged sword for the host during microbial infection. GSDMD-mediated pyroptosis has been verified to contribute to immune defence against different bacterial infections, such as *Shigella*, *Mycobacterium tuberculosis* and *Streptococcus* [36–38]*.* However, GSDMD deficiency results in increased survival in *S. suis*-infected mice [39], indicating the negative role of GSDMD in the host against *S. suis* infection. Our study revealed that *S. suis* induced GSDMD-dependent pyroptosis in macrophages via the release of IL-1β, which is consistent with the finding that *S. suis* induces an inflammatory cytokine storm via the NLRP3 inflammasome [6]. In addition, our results demonstrate that the NLRP3 inflammasome is required for GSDMD-dependent pyroptosis and IL-1β secretion in *S. suis-*infected macrophages. Emerging studies indicate that NLRP3-induced pyroptosis contributes to immune defense against various bacterial and viral infections, including *Mycobacterium tuberculosis* [40], *Staphylococcus pseudintermedius* [11], *Mycobacterium abscessus* [41], respiratory syndrome coronavirus 2 [42] and dengue virus [43]. These studies demonstrate that NLRP3 inflammasome activation plays a critical role in GSDMD-mediated pyroptosis during bacterial infection. A recent study revealed that the critical virulence peptidyl-prolyl isomerase (PrsA) of *S. suis* induces pyroptosis in RAW264.7 cells, but whether other virulence factors are involved in pyroptosis needs to be further studied, and the mechanism by which *S. suis* induces pyroptosis remains unclear. In addition, the gasdermin family plays an important role in bacterial infection-induced pyroptosis. GSDMA has been shown to play an essential role in keratinocyte pyroptosis triggered by *Streptococcus pyogenes* [44]. In addition, Yow et al. recently reported that two pore-forming proteins, GSDMD and GSDME, are crucial in driving anti-*Yersinia* defence [45]. In the present study, we explored the role of GSDMD in *S. suis*-induced macrophage pyroptosis, and whether other gasdermins are involved in *S. suis*-induced macrophage pyroptosis needs to be further investigated.

RACK1, a scaffold protein for kinases and receptors, has multiple biological functions, including signal transduction, tumor cell invasion, angiogenesis, and immune responses [46, 47]. In recent years, RACK1 has been found to regulate microbial infection in hosts, including viruses, bacteria and fungi. RACK1 has been identified as a critical host factor for viral replication, especially during viral infection [48]. A recent study revealed that RACK1 can interact with NLRP3 to mediate inflammasome activation [26]. Our previous study reported that RACK1 is required for *Pasteurella multocida*-induced NLRP3 inflammasome activation [21]. A few studies have shown that RACK1 is involved in pyroptosis. For example, Mycobacterial EST12 has been reported to bind to RACK1 to activate the RACK1-NLRP3-GSDMD pyroptosis-IL-1β pathway [22]. Our study revealed that RACK1 knockdown attenuated *S. suis*-induced pyroptosis, caspase-1 activation, the expression of NLRP3, the maturation of GSDMD, and IL-1β secretion, indicating that RACK1 plays an important role in *S. suis*-induced pyroptosis. However, the exact virulence factor involved in *S. suis*-induced pyroptosis needs to be further studied.

NIMA-related kinase 7 (NEK7), a serine/threonine kinase, is involved in the cell growth cycle. It has been reported that activated cells induce the formation of the NLRP3-NEK7 complex along with caspase-1 activation and ASC speck formation, which triggers pyroptosis by cleaving GSDMD [29, 49]. NEK7-mediated pyroptosis has been reported to be involved in immunological diseases and pathogen infection. Chen et al. reported that NEK7 interacts with NLRP3 to modulate NLRP3 inflammasome activation, thereby modulating pyroptosis in MODE-K cells and DSS-induced chronic colitis in mice [30]. Wang et al. reported that NEK7 promotes pyroptosis via the formation of a complex with NLRP3 upon *Streptococcus pneumoniae* infection [50]. Our data revealed that *S. suis* induced the interaction of NEK7 and NLRP3 and that NEK7 knockdown resulted in reduced IL-1β secretion, ASC oligomerization, the maturation of GSDMD, and cell death, indicating that NEK7 is involved in *S. suis*-induced pyroptosis. Notably, NF-κB signalling reportedly regulates the interaction of NEK7 and NLRP3, thereby modulating pyroptosis in inflammatory bowel disease [30]. Our present study revealed that RACK1 interacted with P65 and that RACK1 knockdown resulted in a significant decrease in the phosphorylation of P65 in *S. suis*-infected macrophages. Wu et al. reported that RACK1 regulates NF-κB signalling via direct interactions between RACK1 and the NF-κB subunits P50 and P65 in diabetic nephropathy [51]. These studies demonstrate that RACK1 mediates the activation of the NF-κB pathway.

In summary, our study revealed the important role of RACK1 and NEK7 in *S. suis*-induced macrophage pyroptosis. First, *S. suis* induces the activation of the NLRP3 inflammasome and caspase-1, resulting in the cleavage of GSDMD, which leads to GSDMD-dependent pyroptosis and IL-1β secretion. Furthermore, RACK1 induced macrophage pyroptosis by mediating the phosphorylation of P65 to promote the expression of NLRP3 and Pro-IL-1β during *S. suis* infection. In addition, *S. suis* induced the formation of the RACK1-NEK7-NLRP3 complex, and NEK7 was involved in macrophage pyroptosis. This study demonstrated that RACK1 and NEK7 are indispensable for *S. suis*-induced pyroptosis and increased our knowledge of the pathogenesis of *S. suis*, which provides a potential strategy for the prevention and treatment of *S. suis*.

## Data Availability

Not applicable.
